# Analyzing HIV/AIDS dynamics with a novel Caputo-Fabrizio fractional order model and optimal control measures

**DOI:** 10.1371/journal.pone.0315850

**Published:** 2024-12-31

**Authors:** Azhar Iqbal Kashif Butt, Muhammad Imran, Komal Azeem, Tariq Ismaeel, Brett Allen McKinney

**Affiliations:** 1 Department of Mathematics and Statistics, College of Science, King Faisal University, Al-Ahsa, Saudi; 2 Tandy School of Computer Science, The University of Tulsa, Tulsa, OK, United States of America; 3 Department of Mathematics, Government College University Lahore, Lahore, Pakistan; Princess Sumaya University for Technology, JORDAN

## Abstract

In this manuscript, we present a novel mathematical model for understanding the dynamics of HIV/AIDS and analyzing optimal control strategies. To capture the disease dynamics, we propose a new Caputo-Fabrizio fractional-order mathematical model denoted as *SE*_*I*_*E*_*U*_*PIATR*, where the exposed class is subdivided into two categories: exposed-identified *E*_*I*_ and exposed-unidentified *E*_*U*_ individuals. Exposed-identified individuals become aware of the disease within three days, while exposed-unidentified individuals remain unaware for more than three days. Simultaneously, we introduce a treatment compartment with post-exposure prophylaxis (PEP), represented as *P*, designed for individuals of the exposed identified class. These individuals initiate treatment upon identification and continue for 28 days, resulting in full recovery from HIV. Additionally, we categorize infectious individuals into two groups: under-treatment individuals, denoted as *T*, and those with fully developed AIDS not receiving antiretroviral therapy (ART) treatment, denoted as *A*. We establish that the proposed model has a unique, bounded, and positive solution, along with other fundamental characteristics. Disease-free and endemic equilibrium points and their associated properties (such as the reproduction number $R_{0}$ and stability analysis) are determined. Sensitivity analysis is performed to assess the impact of parameters on $R_{0}$ and hence on the disease dynamics. Finally, we formulate a fractional optimal control problem to examine strategies for minimizing HIV/AIDS infection while keeping costs at a minimum. We adopt the use of condoms and changes in sexual habits as optimal controls. The numerical results are presented and discussed through graphs.

## 1 Introduction

Acquired Immunodeficiency Syndrome (AIDS) is an infectious disease that compromises the body’s ability to combat infections due to the introduction of the Human Immunodeficiency Virus (HIV) [1]. Discovered in 1981, AIDS has become a global health concern, claiming the lives of approximately 2 million individuals in the year 2009 alone [1, 2]. The World Health Organization (WHO) reports that by the end of 2021, there were 1.5 million new infections and 38.4 million active cases [2]. As of July 2022, the disease has tragically claimed around 40 million lives [3]. About 40.4 million people have died from HIV/AIDS since the epidemic’s start, while 85.6 million individuals have contracted the infection [4]. By the end of 2022, 39 million people worldwide were HIV/AIDS positive. Globally, 0.7% of adults between the ages of 15 and 49 are thought to be HIV/AIDS positive, while the epidemic’s toll varies greatly between nations and areas. With approximately 1 in every 25 adults (3.2%) living with HIV/AIDS and making up more than two-thirds of all HIV-positive individuals globally, the WHO African region continues to be the most severely afflicted [4]. Given the absence of a vaccine and a definitive treatment for AIDS to date, controlling the disease remains a significant challenge. Antiretroviral Therapy (ART) stands as the sole effective treatment for AIDS, enabling individuals to lead longer lives and restore their immune systems [5, 6]. While ART cannot completely eradicate the virus, it can mitigate the severity of the disease, subsequently reducing transmission from HIV-positive individuals to those uninfected. Effective ART also plays a crucial role in preventing the transmission of HIV from an infected mother to her child, thus diminishing the risk of disease transmission [7].

The symptoms and causes of HIV/AIDS vary from person to person and are contingent on the stage of the infection. The early stages are highly infectious, yet individuals may remain unaware until later phases. Early symptoms include influenza-like illnesses such as fever, headache, sore throat, or rash. As the infection progresses and weakens the immune system, additional symptoms such as diarrhea, weight loss, swollen lymph nodes, and coughing may manifest [8, 9]. Untreated, these symptoms can escalate into severe conditions like tuberculosis (TB) and cancer [9]. Sexual intercourse is the primary mode of HIV transmission and accounts for about three-quarters of all global HIV/AIDS infections, classifying HIV/AIDS as a sexually transmitted disease (STD). Although the first recorded case involved transmission between men, the majority of infections result from heterosexual intercourse, and the risk increases with the number of sexual partners, especially through anal intercourse [8, 10]. Another significant transmission mode is from mother to child, occurring during pregnancy, delivery, or breastfeeding. Various risk factors make individuals susceptible to HIV, including the presence of other sexually transmitted infections (STIs) such as herpes, gonorrhea [10], etc. Additionally, engaging in harmful alcohol and drug use during sexual activities, as well as sharing contaminated needles or syringes for drug use or blood transfusion, heightens the risk of contracting HIV [8–10].

The most common method for HIV testing involves detecting antibodies produced by the person’s immune system in response to fighting HIV. Typically, individuals develop antibodies to HIV within less than 28 days [11]. However, during this period, known as the window period, there may be an insufficient quantity of antibodies for HIV to be detectable through tests, especially if the individual exhibits no symptoms. This poses a risk as the infection can be transmitted to others through sexual contact, pregnancy, or breastfeeding during this time [10–12]. It’s important to note that rapid testing is not adequate for detecting HIV/AIDS in babies under 18 months, and virological testing should be conducted after birth or at least at 6 weeks of age [12]. Various strategies can be employed to prevent the spread of HIV/AIDS. Primary among them is the necessity for sexual education, promoting a reduction in the number of sexual partners, and advocating for the use of contraceptive barriers. Male circumcision has also proven to be an effective method for reducing HIV transmission between males and females. Additionally, there should be a focus on the treatment and prevention of sexually transmitted infections (STIs) [13].

In the realm of scientific inquiry, mathematical modeling has played a crucial role in comprehending physical problems and devising solutions [14–19]. Over time, numerous mathematical models have been developed to grasp the dynamics and control strategies of diseases such as coronavirus [18, 19], HBV [20], LSD [15, 21], and HIV/AIDS [22–24]. Scientists and researchers continue to strive towards refining mathematical models of HIV/AIDS by analyzing different biological aspects of the disease and exploring various control strategies to minimize the number of individuals affected. These researchers proposed well-posed mathematical model along with optimal control strategies under the classical integer order derivative [14–21]. Recently, fractional calculus has played a crucial role across various scientific fields, captivating the interest of researchers due to its applications in expressing real-life phenomena [21, 25–28]. The concept of fractional-order derivatives is a generalization of classical-order derivatives, where fractional-order derivatives replace integer-order derivatives [21, 29]. Additionally, the solution to a fractional-order differential equation should converge to the solution of an integer-order differential equation as the derivative order approaches one [30, 31]. Fractional-order models, which can describe memory and hereditary characteristics, are more versatile than classical models. They overcome the limitations (i.e., memory effect and compatibility with real data) of integer-order differential equations when seeking solutions. However, phenomena involving memory and hereditary properties cannot be adequately described by classical integer-order systems [32–35].

In the field of controlling nonlinear systems, various fractional operators, combined with multiple strategies, have been employed, both with and without optimal control strategies. In the context of HIV/AIDS disease, several mathematical models and strategies have been employed to mitigate the disease’s impact. The literature features diverse techniques for preventing HIV/AIDS, including sliding mode control [22] and fuzzy discrete event system approaches [23, 24], feedback control [36, 37], and optimal control [38–42]. In [43, 44], the authors divided the total population into four subclasses (susceptible *S*(*t*), infectives *I*(*t*) (also assumed to be infectious), pre-AIDS patients *P*(*t*), and AIDS patients *A*(*t*)) and five subclasses (susceptible *S*(*t*), infectives *I*(*t*) (also assumed to be infectious), pre-AIDS patients *P*(*t*), treatment *T*(*t*), and AIDS patients *A*(*t*)), respectively. They discussed the vertical transmission of the disease and the stability analysis of the model at both disease-free and endemic equilibrium points. In [44], the bifurcation of the equilibrium points is also included. In both articles, the authors provided numerical simulations along with theoretical results. In [45], the authors proposed an extended SIR Caputo-Fabrizio (CF) fractional mathematical model, incorporating ART treatment and compartments for changes in sexual habits. They proved the fundamental properties of the model, including the existence of a unique, positive, and bounded solution. Additionally, they discussed the stability analysis of equilibrium points under different fractional orders and presented numerical results. In [46], the authors extended the work of [45] by adding sensitivity analysis and by defining a fractional optimal control problem. They proposed treatment, precautions, and changes in sexual habits as optimal control strategies, considering different cases.

Many ordinary and fractional-order mathematical models for HIV/AIDS, both with and without optimal control strategies, have been proposed. However, in this article, we consider more realistic assumptions. We introduce identified exposed and unidentified exposed compartments, a post-exposure prophylaxis (PEP) treatment compartment for identified exposed individuals, and a recovered compartment. We propose a new CF fractional model for HIV/AIDS, prove the fundamental properties of the model, calculate the equilibrium points, and analyze the stability of the model at these equilibrium points. Additionally, a sensitivity analysis is performed to assess the effects of the parameters. Finally, we propose a fractional optimal control problem, introducing condom use and changes in sexual habits as optimal control parameters. The rest of the manuscript is structured as follows: Section 2 discusses the formulation of CF fractional model for HIV/AIDS. Section 3 addresses the fundamental properties of the proposed model, including the existence of a unique, positive, and bounded solution. In Section 4, equilibrium points are determined, and the conditions for the stability of the model at these points are explored. Section 5 conducts sensitivity analysis on the parameters influencing the reproduction number $R_{0}$. The HIV/AIDS fractional model is further enhanced by introducing two controls (i.e., use of condoms and change in sexual habits), and the formulation of the optimization problem is described in Section 6. This section also includes numerical case studies and a detailed discussion. Finally, the study’s findings are concluded in Section 7.

## 2 Model formulation

Mathematical modeling is crucial in understanding disease dynamics within epidemiology. The emergence of the deadly disease HIV/AIDS in the 1980s heightened its significance for researchers. A more realistic model helps decision-makers understand disease dynamics and make informed decisions to control its spread. In this section, we present a mathematical model for HIV/AIDS. We categorize the human population into eight branches: susceptible *S*(*t*), exposed identified *E*_*I*_(*t*), exposed unidentified *E*_*U*_(*t*), post-exposure prophylaxis treatment *P*(*t*), infected *I*(*t*), fully developed AIDS *A*(*t*), treated *T*(*t*), and recovered *R*(*t*). Thus, the total population *N*(*t*) at any time *t* can be written as:

$$\begin{array}{c} N(t)=S\left(t\right)+E_{I}\left(t\right)+E_{U}\left(t\right)+P\left(t\right)+I\left(t\right)+A\left(t\right)+T\left(t\right)+R\left(t\right). \end{array}$$ (1)

**Fig 1:**
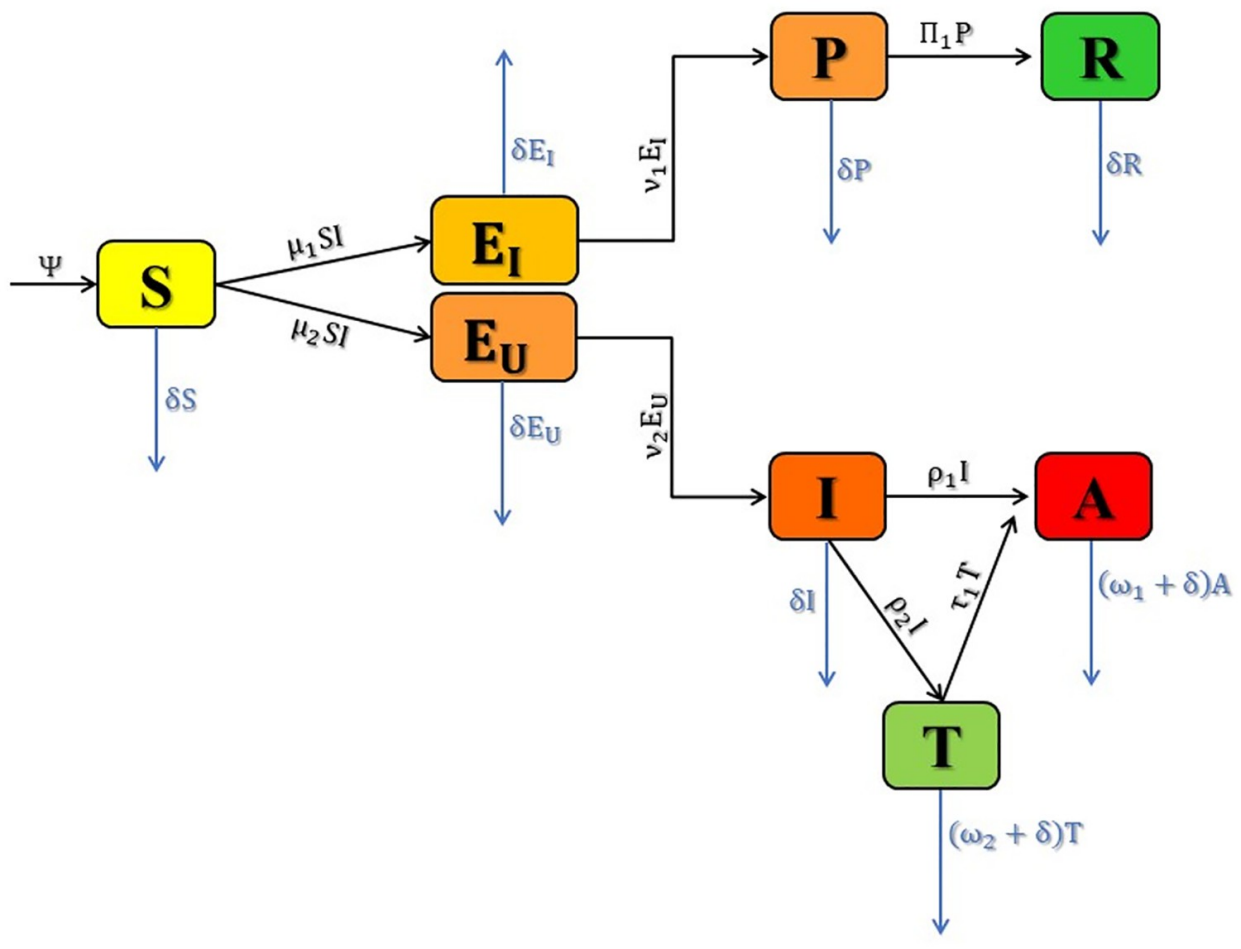
Flow diagram of disease transmissions.

The initial category, referred to as the susceptible branch and denoted by *S*(*t*), includes individuals at a high risk of acquiring HIV/AIDS following contact with an infectious person. Upon interaction with an infectious individual, the susceptible person transitions to the exposed branch, denoted by *E*(*t*), where an infection has occurred but is not yet infectious. Within the exposed branch, two subclasses are identified: exposed identified, denoted by *E*_*I*_(*t*), indicating those diagnosed within three days of exposure, and exposed unidentified, denoted by *E*_*U*_(*t*), representing those infected but unaware of their exposure. As the virus enters the exposed branch, it undergoes constant transformation and reinforcement. Subsequently, the post-exposure prophylaxis (PEP) treatment branch, denoted by *P*(*t*), emerges from the exposed identified class, including individuals receiving initial treatment for 28 days and fully recovering from HIV. The infected branch, denoted by *I*(*t*), arises from the exposed unidentified class and comprises infectious individuals capable of transmitting the virus to other healthy individuals. The next category encompasses those who have fully developed AIDS but are not receiving ART treatment, denoted by *A*(*t*). Following this, the treated class, denoted by *T*(*t*), represents the number of patients undergoing treatment. Finally, *R*(*t*) signifies the number of individuals who have undergone early treatment and are now immune to HIV, categorized as recovered. It is assumed that individuals in the recovered class remain there for the rest of their lives. Moreover, the state variables *S*, *E*_*I*_, *E*_*U*_, *P*, *I*, *A*, *T*, *R* are considered to be continuously differentiable functions of *t* ∈ [0, ∞). Fig 1 illustrates the transmission of HIV/AIDS infection within the population, and a detailed description of the transmission parameters, along with their values, is provided in Table 2.

To thoroughly examine the internal memory effects within the HIV mathematical model, we employ the CF fractional order derivative for a more rational disease forecast, i.e., compatibility of fraction order with real data. Before delving into the fractional model of HIV/AIDS, we provide an overview of some fractional calculus definitions and properties. Caputo and Fabrizio introduced a novel definition for the fractional order derivative, as presented in [47, 48], aimed at eliminating a singularity in its kernel.

**Definition 1** [47, 48] *Let g* ∈ *H*^1^(*u*, *v*) *and α* ∈ (0, 1). *Then the CF fractional derivative of order α is defined as:*

CFDtα(g(t))=Ϝ(α)1-α∫0tg′(x)exp[-α(t-x1-α)]dx,t>0, (2)

*where Ϝ*(*α*) *is a normalization function such that Ϝ*(0) = *Ϝ*(1) = 1.

**Definition 2** [47, 48] *Let α* ∈ (0, 1). *The associated CF fractional integral of order α of a function g is defined by:*

CFItα(g(t))=2(1-α)(2-α)Ϝ(α)g(t)+2α(2-α)Ϝ(α)∫0tg(x)dx,t≥0. (3)

**Theorem 1** [48, 49] *The Laplace transformation of CF fractional order derivative of order α for a function g*(*t*) *is defined as:*

L[CFDtα(g(t))]=sL[g(t)]-g(0)s+α(1-s),s≥0. (4)

**Theorem 2** [48, 49] *The solution of the system*

CFDtα(g(t))=F(t),g(0)=g0, (5)

*of CF fractional differential equations for α* ∈ (0, 1] *is given by*

$$\begin{array}{c} g\left(t\right))=g\left(0\right)+\frac{2}{\left(2-\alpha )Ϝ(\alpha \right)}[\left(1-\alpha \right)F\left(t\right)+\alpha \int_{0}^{t}F\left(s\right)ds]\;\forall \;t>0. \end{array}$$ (6)

Following the disease flow diagram in Fig 1, the CF fractional model for HIV disease is presented as follows:

CFDtαS(t)=Ψ-μ1S(t)I(t)-μ2S(t)I(t)-δS(t), (7a)

CFDtαEI(t)=μ1S(t)I(t)-ν1EI(t)-δEI(t), (7b)

CFDtαEU(t)=μ2S(t)I(t)-ν2EU(t)-δEU(t), (7c)

CFDtαP(t)=ν1EI(t)-Π1P(t)-δP(t), (7d)

CFDtαI(t)=ν2EU(t)-ρ1I(t)-ρ2I(t)-δI(t), (7e)

CFDtαA(t)=ρ1I(t)+τ1T(t)-(ω1+δ)A(t), (7f)

CFDtαT(t)=ρ2I(t)-τ1T(t)-(ω2+δ)T(t), (7g)

CFDtαR(t)=Π1P(t)-δR(t), (7h)

with following conditions:

$$\begin{array}{c} S\left(0\right)>0,\;E_{I}\left(0\right)\geq 0,\;E_{U}\left(0\right)\geq 0,\;P\left(0\right)\geq 0,\;I\left(0\right)\geq 0,\;A\left(0\right)\geq 0,\;T\left(0\right)\geq 0,\;R\left(0\right)\geq 0. \end{array}$$ (7i)

All parameters utilized in the model are presumed to be non-negative constants, each serving a specific purpose as described below.

- Ψ denotes the birth rate of susceptible individuals entering the population.
- *μ*_1_ signifies the contact rate between susceptible and infectious individuals, leading to entry into the exposed identified class.
- *μ*_2_ represents the transmission rate between susceptible and exposed unidentified classes following physical contact with infectious individuals.
- *ν*_1_ denotes the rate at which exposed identified individuals exit the class and move into *P*(*t*).
- *ν*_2_ indicates the number of exposed unidentified individuals transitioning per unit time to the infectious class.
- Π_1_ signifies the rate at which individuals receiving post-exposure prophylaxis (PEP) treatment enter the recovered class.
- *ρ*_1_ represents the rate at which individuals exit the infectious class and progress to individuals with fully developed AIDS per unit time.
- *ρ*_2_ indicates the rate at which infectious individuals receive antiretroviral therapy (ART) treatment.
- *τ*_1_ represents the rate at which individuals lacking sufficient treatment leave the treated class and enter the AIDS class.
- *δ* denotes the natural death rate.
- *ω*_1_ and *ω*_2_ represent the death rates due to disease for individuals in the *A*(*t*) and *T*(*t*) classes, respectively.

The proposed model (7) is an autonomous system of differential equations; we can write it in the compact form as follows.

CFDtαZ(t)=G(Z(t)),Z(0)=Z0, (8)

where

$$\begin{array}{c} Z\left(t\right)={\left(S\left(t\right),E_{I}\left(t\right),E_{U}\left(t\right),P\left(t\right),I\left(t\right),A\left(t\right),T\left(t\right),R\left(t\right)\right)}^{T}\in R^{8}, \end{array}$$

and

$$\begin{array}{c} Z_{0}={\left(S\left(0\right),E_{I}\left(0\right),E_{U}\left(0\right),P\left(0\right),I\left(0\right),A\left(0\right),T\left(0\right),R\left(0\right)\right)}^{T}, \end{array}$$

and

$$\begin{array}{c} G\left(Z\left(t\right)\right)=(\begin{array}{c} G_{1}\\ G_{2}\\ G_{3}\\ G_{4}\\ G_{5}\\ G_{6}\\ G_{7}\\ G_{8} \end{array})=(\begin{array}{c} \Psi -\mu_{1}S\left(t\right)I\left(t\right)-\mu_{2}S\left(t\right)I\left(t\right)-\delta S\left(t\right)\\ \mu_{1}S\left(t\right)I\left(t\right)-\nu_{1}E_{I}\left(t\right)-\delta E_{I}\left(t\right)\\ \mu_{2}S\left(t\right)I\left(t\right)-\nu_{2}E_{U}\left(t\right)-\delta E_{U}\left(t\right)\\ \nu_{1}E_{I}\left(t\right)-\Pi_{1}P\left(t\right)-\delta P\left(t\right)\\ \nu_{2}E_{U}\left(t\right)-\rho_{1}I\left(t\right)-\rho_{2}I\left(t\right)-\delta I\left(t\right)\\ \rho_{1}I\left(t\right)+\tau_{1}T\left(t\right)-\left(\omega_{1}+\delta \right)A\left(t\right)\\ \rho_{2}I\left(t\right)-\tau_{1}T\left(t\right)-\left(\omega_{2}+\delta \right)T\left(t\right)\\ \Pi_{1}P\left(t\right)-\delta R\left(t\right) \end{array}). \end{array}$$

## 3 Fundamental properties

We seek to prove the essential characteristics of the proposed epidemic model (8) in this section, such as the presence of a unique, positive, and bounded solution. We prove the existence and uniqueness of the solutions using the well-known results from fractional calculus. Applying Laplace transformation properties specific to the CF fractional operator will be instrumental in proving the positivity and boundedness of the solutions. This not only underscores the accuracy and reliability of the model but also attests to the robustness of the system of differential equations.

### 3.1 Establishment of a unique solution

We implement the well-known theorems from functional analysis to establish the existence of a unique solution. Picard’s successive iterative approach along with fixed-point theory is used to support the proof of the stated theorems.

**Theorem 3** *The function* G(Z(t)) *given in* (8) *is Lipschitz continuous on C*^1^[0, *T*_*f*_].

**Proof:** Let V be any convex subset,

$$\begin{array}{c} V\subseteq D=\{Z\left(t\right)|0\leq t\leq T_{f},Z\in R_{+}^{8}\}. \end{array}$$ (9)

If $Z_{1},Z_{2}\in V$ then by Mean Value Theorem there exists some $\xi \in (Z_{1},Z_{2})$ such that

$$\begin{array}{c} \begin{array}{cc} |G\left(Z_{1}\left(t\right)\right)-G\left(Z_{2}\left(t\right)\right)| & =|G^{\prime }\left(\xi \left(t\right)\right)\left(Z_{1}\left(t\right)-Z_{2}\left(t\right)\right)|,\\ & \leq ∥G^{\prime }\left(\xi \left(t\right)\right){∥}_{\infty }{∥Z_{1}-Z_{2}∥}_{\infty },\;\left(usingCauchy-Schiwarzproprty\right). \end{array} \end{array}$$

Since all the state variables are assumed to be continuously differentiable, we can say $G\in C^{1}\left[0,T_{f}\right]$, and for every convex subset, there exists a positive constant C>0 such that

$$\begin{array}{c} ∥G\prime \left(\xi \right){∥}_{\infty }\leq C. \end{array}$$

Hence,

$$\begin{array}{c} |G\left(Z_{1}\left(t\right)\right)-G\left(Z_{2}\left(t\right)\right)|\leq C∥Z_{1}-Z_{2}{∥}_{\infty }, \end{array}$$

or

$$\begin{array}{c} \underset{t\in [0,T_{f}]}{\text{sup}}|G\left(Z_{1}\left(t\right)\right)-G\left(Z_{2}\left(t\right)\right)|\leq C∥Z_{1}-Z_{2}{∥}_{\infty }. \end{array}$$

This implies the following inequality:

$$\begin{array}{c} ∥G\left(Z_{1}\right)-G\left(Z_{2}\right){∥}_{\infty }\leq C∥Z_{1}-Z_{2}{∥∥}_{\infty }. \end{array}$$

Thus, G(Z(t)) is Lipschitz. □

**Theorem 4** *Suppose that the function* G(Z(t)) *satisfies the Lipschitz condition*

$$\begin{array}{c} ∥G\left(Z_{2}\right)-G\left(Z_{1}\right){∥}_{\infty }\leq C{∥Z_{1}-Z_{2}∥}_{\infty }, \end{array}$$

*then the proposed model* (8) *has a unique solution for*

$$\begin{array}{c} \frac{2C}{\left(2-\alpha )Ϝ(\alpha \right)}\left[\left(1-\alpha \right)+\alpha T_{f}\right]<1. \end{array}$$

**Proof:** The function Z(t) is a solution of the problem (8) if and only if it satisfies the equation

$$\begin{array}{c} Z\left(t\right)=Z_{0}+\frac{2}{\left(2-\alpha )Ϝ(\alpha \right)}[\left(1-\alpha \right)G\left(Z\left(t\right)\right)+\alpha \int_{0}^{t}G\left(Z\left(x\right)\right)dx]. \end{array}$$ (10)

Suppose Z(t) be the solution of the Eq (8). We apply the CF integral (2) to Eq (8), i.e.,

CFItα[CFDtα(Z(t))]=CFItαG(Z(t)), (11)

and expand it to get the solution given by Eq (10).

For the converse implication, we define a sequence of functions $Z_{n}$ that converges to the solution of Eq (10) with Picard successive iterations:

$$\begin{array}{c} \begin{array}{cc} Z_{n}\left(t\right) & =Z_{0}+\frac{2}{\left(2-\alpha )Ϝ(\alpha \right)}[\left(1-\alpha \right)G\left(Z_{n-1}\left(t\right)\right)+\alpha \int_{0}^{t}G\left(Z_{n-1}\left(x\right)\right)dx],\;n=1,2,..\\ \text{with}\;Z(t_{0}) & =Z_{0}. \end{array} \end{array}$$ (12)

Consider

$$\begin{array}{cc} \left|Z_{n}\left(t\right)-Z_{n-1}\left(t\right)\right| & =|\frac{2(1-\alpha )}{\left(2-\alpha )Ϝ(\alpha \right)}[G\left(Z_{n-1}\left(t\right)\right)-G\left(Z_{n-2}\left(t\right)\right)]\\ & +\frac{2\alpha }{\left(2-\alpha )Ϝ(\alpha \right)}\int_{0}^{t}[G\left(Z_{n-1}\left(x\right)\right)-G\left(Z_{n-2}\left(x\right)\right)]dx|,\\ & \leq \frac{2(1-\alpha )}{\left(2-\alpha )Ϝ(\alpha \right)}|G\left(Z_{n-1}\left(t\right)\right)-G\left(Z_{n-2}\left(t\right)\right)|\\ & +\frac{2\alpha }{\left(2-\alpha )Ϝ(\alpha \right)}\int_{0}^{t}|G\left(Z_{n-1}\left(x\right)\right)-G\left(Z_{n-2}\left(x\right)\right)|dx. \end{array}$$

Since G(Z(t)) is Lipschitz, therefore,

$$\begin{array}{cc} |Z_{n}\left(t\right)-Z_{n-1}\left(t\right)|\leq & \frac{2(1-\alpha )}{\left(2-\alpha )Ϝ(\alpha \right)}C\underset{t\in [0,T_{f}]}{\text{sup}}|Z_{n-1}\left(t\right)-Z_{n-2}\left(t\right)|\\ & +\frac{2\alpha }{\left(2-\alpha )Ϝ(\alpha \right)}\int_{0}^{t}C\underset{x\in [0,T_{f}]}{\text{sup}}|Z_{n-1}\left(x\right)-Z_{n-2}\left(x\right)|dx,\\ \leq & [\frac{2(1-\alpha )C}{\left(2-\alpha )Ϝ(\alpha \right)}+\frac{2\alpha C}{\left(2-\alpha )Ϝ(\alpha \right)}]{∥Z_{n-1}-Z_{n-2}∥}_{\infty }, \end{array}$$

and

$$\begin{array}{cc} \underset{t\in [0,T_{f}]}{\text{sup}}|Z_{n}\left(t\right)-Z_{n-1}\left(t\right)| & \leq [\frac{2(1-\alpha )C}{\left(2-\alpha )Ϝ(\alpha \right)}+\frac{2\alpha C}{\left(2-\alpha )Ϝ(\alpha \right)}]{∥Z_{n-1}-Z_{n-2}∥}_{\infty }. \end{array}$$

If $K=\frac{2C}{\left(2-\alpha )Ϝ(\alpha \right)}[\left(1-\alpha \right)+\alpha T_{f}]<1,$ then

$$\begin{array}{cc} ∥Z_{n}-Z_{n-1}{∥}_{\infty } & \leq K∥Z_{n-1}-Z_{n-2}{∥}_{\infty }, \end{array}$$

or

$$\begin{array}{c} d\left(Z_{n},Z_{n-1}\right)\leq Kd\left(Z_{n-1},Z_{n-2}\right), \end{array}$$

and hence, the sequence (12) is a contractive sequence. Therefore, it is a Cauchy sequence.

Let m*, n* ∈ **N** and n* > m*

$$\begin{array}{cc} |Z_{n^{*}}-Z_{m^{*}}| & =|Z_{n^{*}}-Z_{n^{*}-1}+Z_{n^{*}-1}-Z_{n^{*}-2}+Z_{n^{*}-2}...-Z_{m^{*}+1}+Z_{m^{*}+1}-Z_{m^{*}}|,\\ & \leq |Z_{n^{*}}-Z_{n^{*}-1}\left|+\right|Z_{n^{*}-1}-Z_{n^{*}-2}\left|+...+\right|Z_{m^{*}+1}-Z_{m^{*}}|,\\ & \leq K^{n^{*}-1}|Z_{1}-Z_{0}|+K^{n^{*}-2}|Z_{1}-Z_{0}|+...+K^{m^{*}}\left|Z_{1}-Z_{0}\right|,\\ & \leq \left[K^{n^{*}-1}+K^{n^{*}-2}+...+K^{m^{*}}\right]\left|Z_{1}-Z_{0}\right|. \end{array}$$

With 0 < K < 1, the following inequality is obvious.

$$\begin{array}{c} |Z_{n^{*}}-Z_{m^{*}}|\leq K^{m^{*}}\frac{1-K^{n^{*}-m^{*}}}{1-K}|Z_{1}-Z_{0}|\leq \frac{K^{m^{*}}}{1-K}\left|Z_{1}-Z_{0}\right|. \end{array}$$

Since $K^{m^{*}}\to 0$ as m* → ∞, the sequence $\left(Z_{n^{*}}\right)$ is Cauchy, it always converges.

Suppose $\lim_{n\to \infty }Z_{n^{*}}=Z$, then

$$\begin{array}{c} \lim_{n\to \infty }Z_{n^{*}}\left(t\right)=Z\left(t\right)=Z_{0}+\frac{2}{\left(2-\alpha )Ϝ(\alpha \right)}[\left(1-\alpha \right)G\left(Z\left(t\right)\right)+\alpha \int_{0}^{t}G\left(Z\left(x\right)\right)dx]. \end{array}$$ (13)

Thus, the Eq (13) is the required solution.

We then establish the solution’s uniqueness. Suppose, on the contrary, if the sequence $\left(Z_{n}\right)$ converges to two distinct limits, $\tilde{Z}$ and $\bar{Z}$, then there exists some *m*_1_, *m*_2_ ∈ **N** such that

$$\begin{array}{cc} |Z_{n}-\tilde{Z}| & <\epsilon_{1}\;for\;m_{1},\\ |Z_{n}-\bar{Z}| & <\epsilon_{2}\;for\;m_{2}. \end{array}$$

Let *m* = max{*m*_1_, *m*_2_},

$$\begin{array}{cc} |\tilde{Z}-\bar{Z}| & =|\tilde{Z}-Z_{n}+Z_{n}-\bar{Z}\left|\leq \right|\tilde{Z}-Z_{n}\left|+\right|Z_{n}-\bar{Z}|<\epsilon_{1}+\epsilon_{2}=\epsilon . \end{array}$$

This implies that $\tilde{Z}=\bar{Z}$, which is a contradiction.

### 3.2 Bounded and positive solution

When dealing with a biological model involving the human population, ensuring that the solutions are both bounded and positive is crucial. As a result, in the upcoming theorems, we prove that the solution of the proposed model (7) stays within a feasible region and maintains its bounded and positive nature.

**Theorem 5** *The solution* Z(t) *of the CF fractional HIV/AIDS model* (7) *is bounded for all t* > 0.

**Proof:** The total population *N*(*t*) at any time *t* is defined as follows:

$$\begin{array}{c} N\left(t\right)=S\left(t\right)+E_{I}\left(t\right)+E_{U}\left(t\right)+P\left(t\right)+I\left(t\right)+A\left(t\right)+T\left(t\right)+R\left(t\right). \end{array}$$ (14)

Applying the CF derivative on both sides of Eq (14) and then substituting the right-hand sides of system (7), we obtain the following:

CFDtαN(t)=Ψ-ω1A(t)-ω2T(t)-δN(t),≤Ψ-δN(t).

Application of Laplace transform yields us

$$\begin{array}{cc} \frac{sN(s)}{s+\alpha (1-s)}+\delta N\left(s\right) & \leq \frac{\Psi }{s}+\frac{N(0)}{s+\alpha (1-s)}, \end{array}$$

which can be simplified to get the expression for *N*(*s*), i.e.,

$$\begin{array}{c} N(s)\leq \frac{\Psi \left(1-\alpha \right)+\Psi s^{-1}\alpha +N\left(0\right)}{(1+\delta -\delta \alpha )s+\delta \alpha }. \end{array}$$

Re-arrange the terms to obtain the following inequality.

$$\begin{array}{cc} N(s) & \leq \frac{\Psi (1-\alpha )}{1+\delta -\delta \alpha }[\frac{s^{0}}{s-[-\frac{\delta \alpha }{1+\delta -\delta \alpha }]}]+\frac{\Psi \alpha }{1+\delta -\delta \alpha }[\frac{s^{1-2}}{s-[-\frac{\delta \alpha }{1+\delta -\delta \alpha }]}]\\ & +\frac{N(0)}{1+\delta -\delta \alpha }[\frac{s^{0}}{s-[-\frac{\delta \alpha }{1+\delta -\delta \alpha }]}]. \end{array}$$

We apply the inverse Laplace transform to obtain an upper bound for *N*(*t*) as follows:

$$\begin{array}{c} N\left(t\right)\leq \frac{\Psi (1-\alpha )}{1+\delta -\delta \alpha }E_{1,1}(-\varphi t)+\frac{\Psi \alpha }{1+\delta -\delta \alpha }tE_{1,2}(-\varphi t)+\frac{N(0)}{1+\delta -\delta \alpha }E_{1,1}(-\varphi t), \end{array}$$

where $\phi =\frac{\delta \alpha }{1+\delta -\delta \alpha }$ and we have used the following Laplace transform of Mittag-Leffler function *E*_*τ*,*γ*_, *τ*, *γ* > 0.

$$\begin{array}{c} L\left\{t^{\gamma -1}E_{\tau ,\gamma }\left(\pm yt^{\tau }\right)\right\}=\frac{s^{\tau -\gamma }}{s^{\tau }\mp y}. \end{array}$$

The Mittag-Leffler function always shows an asymptotic behavior. Thus, it can be easily seen that $N\left(t\right)\leq \frac{\Psi }{\delta }$ as *t* → ∞. Thus, the *N*(*t*) and all other state variables of the proposed HIV/AIDS model (7) are bounded.

**Theorem 6** *The solution* Z(t) *of the proposed CF fractional model* (7) *is positive for non-negative initial conditions for all t* ≥ 0.

**Proof:** Considering the first equation of the model (2), i.e.,

CFDtαS(t)=Ψ-(μ1I(t)+μ2I(t)+δ)S(t).

All the state variables of model (7) are bounded; we can assume that max{*μ*_1_*I*(*t*) + *μ*_2_*I*(*t*) + *δ*}≤*m* for some positive *m*. Then,

CFDtαS(t)≥-mS(t).

Application of Laplace transform yields us:

$$\begin{array}{c} (\frac{sL\{S(t)\}-S(0)}{s+\alpha (1-s)})\geq -mL\left\{S\left(t\right)\right\}, \end{array}$$

which can be solved to obtain:

$$\begin{array}{c} L\left\{S\left(t\right)\right\}\geq S\left(0\right)(\frac{1}{1+m-m\alpha })(\frac{1}{s-(-\frac{m\alpha }{1+m-m\alpha })}). \end{array}$$

Now, we apply the inverse Laplace transform to obtain:

$$\begin{array}{cc} S(t) & \geq S\left(0\right)(\frac{1}{1+m-m\alpha })(E_{1,1}\left(-\tilde{\varphi }t\right)). \end{array}$$ (15)

where $\tilde{\phi }=\frac{m\alpha }{1+m-m\alpha }$. When both *τ* = *γ* = 1, the Mittag-Leffler function *E*_*τ*,*γ*_ is equivalent to the exponential function so that we can write *E*_1,1_(*y*) = exp(*y*). Then,

$$\begin{array}{c} S\left(t\right)\geq S\left(0\right)(\frac{1}{1+m-m\alpha })exp\left(-\tilde{\varphi }t\right). \end{array}$$ (16)

Since, *S*(0)>0 and $0\leq \frac{exp(-\tilde{\phi }t)}{1+m-m\alpha }\leq 1$, both the quantities on the right-hand side are non-negative. This implies that *S*(*t*) is positive for all *t* ≥ 0. Similarly, we can prove the positivity of the other state variables *E*_*I*_(*t*), *E*_*U*_(*t*), *P*(*t*), *I*(*t*), *A*(*t*), *T*(*t*), *R*(*t*), for all *t* ≥ 0.

## 4 Stability analysis at equilibrium points

Two main equilibrium points exist for an epidemic model. These are disease-free equilibrium (DFE) and endemic equilibrium (EE) points. To find the equilibrium points, we set the rate of change of all the state functions equal to zero, i.e., we set

CFDtαZ(t)=0,

in Eq (7) and put *I* = 0 in the resulting equations to obtain the following DFE point.

$$\begin{array}{c} Q^{0}=\left(\frac{\Psi }{\delta },0,0,0,0,0,0,0\right), \end{array}$$ (17)

and when *I* ≠ 0, we obtain the following EE point.

$$\begin{array}{c} Q^{1}=(S^{1},E_{I}^{1},E_{U}^{1},P^{1},I^{1},A^{1},T^{1},R^{1}), \end{array}$$ (18)

where

$$\begin{array}{cc} S^{1} & =\frac{\Psi }{k_{8}},\;E_{I}^{1}=\frac{\mu_{1}\Psi I^{1}}{k_{1}k_{8}},\;E_{U}^{1}=\frac{\mu_{2}\Psi I^{1}}{k_{2}k_{8}},\;P^{1}=\frac{\nu_{1}\mu_{1}\Psi I^{1}}{k_{1}k_{3}k_{8}},\\ I^{1} & =\frac{\nu_{2}\mu_{2}\Psi }{k_{2}k_{4}k_{7}}-\frac{\delta }{k_{7}},\;A^{1}=(\frac{\rho_{1}k_{6}+\tau_{1}\rho_{2}}{k_{6}k_{5}})I^{1},\;T^{1}=\frac{\rho_{2}I^{1}}{k_{6}},\;R^{1}=\frac{\Pi_{1}\nu_{1}\mu_{1}\Psi I^{1}}{\delta k_{1}k_{3}k_{8}}, \end{array}$$

and

$$\begin{array}{c} k_{1}=\nu_{1}+\delta ,\;k_{2}=\nu_{2}+\delta ,\;k_{3}=\Pi_{1}+\delta ,\;k_{4}=\rho_{1}+\rho_{2}+\delta ,\\ k_{5}=\omega_{1}+\delta ,\;k_{6}=\tau_{1}+\omega_{2}+\delta ,\;k_{7}=\mu_{1}+\mu_{2},\;k_{8}=\mu_{1}I^{1}+\mu_{2}I^{1}+\delta . \end{array}$$

### 4.1 Reproduction number

In the field of epidemiology, the term reproduction number pertains to the average number of secondary cases produced by a single infectious individual within a susceptible population. This measure is also called the basic reproduction number and is denoted by $R_{0}$. To determine this fundamental value, we employ the next-generation matrix method, initially introduced by Diekmann, Heesterbeek, and Metz in 1990 [50]. The basic reproduction number is computed by solving the spectral norm of *FV*^−1^ at DFE point. Here, *F* represents the Jacobian matrix of secondary cases for disease classes and *V* signifies the Jacobian matrix of the remaining terms in the equation for disease classes, i.e.,

$$\begin{array}{c} \begin{array}{cc} F & =(\frac{\partial F_{n}}{\partial \chi_{n}}){|}_{Q^{0}}\;\text{and}\;V=(\frac{\partial V_{n}}{\partial \chi_{n}}){|}_{Q^{0}};\;n=1,2,3,4,5,6, \end{array} \end{array}$$

where

$$\begin{array}{c} \left(\chi_{1},\chi_{2},\chi_{3},\chi_{4},\chi_{5},\chi_{6}\right)=\left(E_{I},E_{U},P,I,A,T\right), \end{array}$$

and

$$\begin{array}{c} F=(\begin{array}{c} \mu_{1}SI\\ \mu_{2}SI\\ 0\\ 0\\ 0\\ 0 \end{array}),\;\;\;V=(\begin{array}{c} \nu_{1}E_{I}+\delta E_{I}\\ \nu_{2}E_{U}+\delta E_{U}\\ -\nu_{1}E_{I}+\Pi_{1}P+\delta P\\ -\nu_{2}E_{U}+\rho_{1}I+\rho_{2}I+\delta I\\ -\rho_{1}I-\tau_{1}T+\left(\omega_{1}+\delta \right)A\\ -\rho_{2}I+\tau_{1}T+\left(\omega_{2}+\delta \right)T \end{array}). \end{array}$$

We compute the spectral radius of the product matrix *FV*^−1^ to obtain the following reproduction number.

$$\begin{array}{cc} R_{0} & =\frac{\mu_{2}\nu_{2}\Psi }{\delta \left(\rho_{1}+\rho_{2}+\delta \right)\left(\nu_{2}+\delta \right)}. \end{array}$$ (19)

The HIV/AIDS will be epidemic provided $R_{0}$ is greater than one.

### 4.2 Local and global stability

In this section, we analyze the stability of the proposed HIV/AIDS CF fractional model (7) at the equilibrium points. We theoretically use $R_{0}$ to discuss local and global stabilities for the given system of equations at both equilibrium points. We adopt the Jacobian approach to prove local stability, and for global stability, we adopt the Castillo Chavez and the Lyapunov approach, respectively.

**Theorem 7** *The proposed CF fractional model* (7) *is locally asymptotically stable (LAS) at DFE point* $Q^{0}$ for $R_{0}<1$ *and unstable otherwise*.

**Proof:** The Jacobian matrix is evaluated at $Q^{0}$ to give

$$\begin{array}{c} \begin{array}{cc} J_{Q^{0}} & =(\begin{array}{cccccccc} -\delta & 0 & 0 & 0 & -\frac{(u_{1}+u_{2})\Psi }{\delta } & 0 & 0 & 0\\ 0 & -k_{1} & 0 & 0 & \frac{\mu_{1}\Psi }{\delta } & 0 & 0 & 0\\ 0 & 0 & -k_{2} & 0 & \frac{\mu_{2}\Psi }{\delta } & 0 & 0 & 0\\ 0 & \nu_{1} & 0 & -k_{3} & 0 & 0 & 0 & 0\\ 0 & 0 & \nu_{2} & 0 & -k_{4} & 0 & 0 & 0\\ 0 & 0 & 0 & 0 & \rho_{1} & -k_{5} & \tau_{1} & 0\\ 0 & 0 & 0 & 0 & \rho_{2} & 0 & -k_{6} & 0\\ 0 & 0 & 0 & \Pi_{1} & 0 & 0 & 0 & -\delta \end{array}). \end{array} \end{array}$$ (20)

Eigenvalues of the Jacobian matrix (20) are computed to give:

$$\begin{array}{c} \begin{array}{cc} \lambda_{1} & =-k_{5},\\ \lambda_{2} & =-k_{3},\\ \lambda_{3} & =-k_{1},\\ \lambda_{4} & =-k_{2},\\ \lambda_{5} & =-k_{6},\\ \lambda_{6} & =-\delta ,\\ \lambda_{7} & =-\delta ,\\ \lambda_{8} & =k_{4}\left(R_{0}-1\right). \end{array} \end{array}$$ (21)

It is evident from the Eq (21) that all the eigenvalues are negative when $R_{0}<1$ and vice versa. Thus, the proposed CF-fractional model (7) is locally asymptotically stable when $R_{0}<1$ and unstable otherwise.

To determine the global stability of the proposed model at $Q^{0}$, Castillo-Chavez approach [51] is adopted and we rewrite the equations of model (7) in the form:

CFDtαN=N(N,D),CFDtαD=D(N,D),D(N,0)=0,

where N represents uninfected (i.e., susceptible and recovered) and D represents the infected population, i.e., *E*_*I*_, *E*_*U*_, *P*, *I*, *A*, *T*.

The following conditions should be satisfied to prove the proposed model’s globally asymptotically stable (GAS) at DFE point.

(K1)CFDtαN=N(N0,0)=0,N0isGAS, (22)

(K2)CFDtαD=D(N,D)=MD-D¯(N,D),D¯(N,D)≥0, (23)

where, $M=\partial_{D}D\left(N_{0},0\right)$ denotes an M-matrix.

**Theorem 8** *If* $R_{0}<1$ , *then the system of* Eq (7) *is globally asymptotically stable (GAS) at* $Q^{0}=\left(N_{0},0\right)$ *if K*1 *and K*2 *are fulfilled*.

**Proof:** Let N=(S,R) represent uninfected individuals and $D=(E_{I},E_{U},P,I,A,T)$ represent people with infection. As we know that $Q^{0}=\left(N_{0},0\right)$ is the disease-free equilibrium point. So,

CFDtαN=N(N,D)=(Ψ-μ1SI-μ2SI-δSΠ1P-δR) (24)

At $Q^{0},N\left(N^{0},0\right)=0$, i.e.,

CFDtαN=(Ψ-μ1S0I0-μ2S0I0-δS0Π1P0-δR0)=(00). (25)

From Eq (25) as $t\to \infty ,N\to N^{0}$. Therefore, $N^{0}$ is GAS. Now

$$\begin{array}{cc} MD-\bar{D}\left(N,D\right) & =[\begin{array}{cccccc} -k_{1} & 0 & 0 & \mu_{1}S^{0} & 0 & 0\\ 0 & -k_{2} & 0 & \mu_{2}S^{0} & 0 & 0\\ 0 & 0 & -k_{3} & 0 & 0 & 0\\ 0 & \nu_{2} & 0 & -k_{4} & 0 & 0\\ 0 & 0 & 0 & \rho_{1} & -k_{5} & \tau_{1}\\ 0 & 0 & 0 & \rho_{2} & 0 & -k_{6} \end{array}][\begin{array}{c} E_{I}\\ E_{U}\\ P\\ I\\ A\\ T \end{array}]-[\begin{array}{c} \mu_{1}K\\ \mu_{2}K\\ 0\\ 0\\ 0\\ 0 \end{array}], \end{array}$$

where

$$\begin{array}{c} \begin{array}{cc} M & =[\begin{array}{cccccc} -k_{1} & 0 & 0 & \mu_{1}S^{0} & 0 & 0\\ 0 & -k_{2} & 0 & \mu_{2}S^{0} & 0 & 0\\ 0 & 0 & -k_{3} & 0 & 0 & 0\\ 0 & \nu_{2} & 0 & -k_{4} & 0 & 0\\ 0 & 0 & 0 & \rho_{1} & -k_{5} & \tau_{1}\\ 0 & 0 & 0 & \rho_{2} & 0 & -k_{6} \end{array}],D=[\begin{array}{c} E_{I}\\ E_{U}\\ P\\ I\\ A\\ T \end{array}],\;\bar{D}\left(N,D\right)=[\begin{array}{c} \mu_{1}K\\ \mu_{2}K\\ 0\\ 0\\ 0\\ 0 \end{array}], \end{array} \end{array}$$

and $K=I(S^{0}-S)$.

The M is an M-matrix, and *S*, *E*_*I*_, *E*_*U*_, *P*, *I*, *A*, *T*, *R* ≤ *S*^0^ at DFE point. Therefore, $\bar{D}\left(N,D\right)\geq 0$. Thus, the DFE point $Q^{0}$ is GAS.

**Theorem 9** If $R_{0}>1$, then the system of Eq (7) at EE is GAS otherwise unstable.

**Proof:** Consider a Volterra type Lyapnouv function defined as follows:

$$\begin{array}{cc} V(S,E_{I},E_{U},P,I,A,T,R) & =[S-S^{1}-S^{1}\text{log}\frac{S}{S^{1}}]+[E_{I}-E_{I}^{1}-E_{I}^{1}\text{log}\frac{E_{I}}{E_{I}^{1}}]\\ & +[E_{U}-E_{U}^{1}-E_{U}^{1}\text{log}\frac{E_{U}}{E_{U}^{1}}]+[P-P^{1}-P^{1}\text{log}\frac{P}{P^{1}}]\\ & +[I-I^{1}-I^{1}\text{log}\frac{I}{I^{1}}]+[A-A^{1}-A^{1}\text{log}\frac{A}{A^{1}}]\\ & +[T-T^{1}-T^{1}\text{log}\frac{T}{T^{1}}]+[R-R^{1}-R^{1}\text{log}\frac{R}{R^{1}}], \end{array}$$

where $\left(S^{1},E_{I}^{1},E_{U}^{1},P^{1},I^{1},A^{1},T^{1},R^{1}\right)$ represents the EE point of the proposed model (7). Applying the CF derivative to time *t* and then simplifying, we get:

CFDtαV(t)=[S-S1S]CFDtαS(t)+[EI-EI1EI]CFDtαEI(t)+[EU-EU1EU]CFDtαEU(t)+[P-P1P]CFDtαP(t)+[I-I1I]CFDtαI(t)+[A-A1A]CFDtαA(t)+[T-T1T]CFDtαT(t)+[R-R1R]CFDtαR(t).

Using equations of the system (7), we obtain the following.

CFDtαV(t)=[S-S1S][Ψ-μ1SI-μ2SI-δS]+[EI-EI1EI][μ1SI-ν1EI-δEI]+[EU-EU1EU][μ2SI-ν2EU-δEU]+[P-P1P][ν1EI-Π1P-δP]+[I-I1I][ν2EU-ρ1I-ρ2I-δI(t)]+[A-A1A][ρ1I+τ1T-(ω1+δ)A]+[T-T1T][ρ2I-τ1T-(ω2+δ)T]+[R-R1R][Π1P-δR].

After simplifying and re-arranging the terms, we write the above expression in the following form:

CFDtαV(t)=q1-q2,

where

$$\begin{array}{cc} q_{1} & =\Psi +[\mu_{1}I+\mu_{2}I+\delta ]\frac{{\left(S^{1}\right)}^{2}}{S}+[\mu_{1}I+\mu_{2}I]S+[\nu_{1}+\delta ]\frac{{\left(E_{I}^{1}\right)}^{2}}{E_{I}}+\nu_{1}E_{I}\\ & +[\nu_{2}+\delta ]\frac{{\left(E_{U}^{1}\right)}^{2}}{E_{U}}+\nu_{2}E_{U}+[\Pi_{1}+\delta ]\frac{{\left(P^{1}\right)}^{2}}{P}+\Pi_{1}P+[\rho_{1}+\rho_{2}+\delta ]\frac{{\left(I^{1}\right)}^{2}}{I}\\ & +\left(\rho_{1}+\rho_{2}\right)I+[\omega_{1}+\delta ]\frac{{\left(A^{1}\right)}^{2}}{A}+\tau_{1}T+[\tau_{1}+\delta ]\frac{{\left(T^{1}\right)}^{2}}{T}+\delta \frac{{\left(R^{1}\right)}^{2}}{R}, \end{array}$$

and

$$\begin{array}{cc} q_{2} & =\Psi \frac{S^{1}}{S}+[\mu_{1}I+\mu_{2}I+\delta ]\frac{{\left(S-S^{1}\right)}^{2}}{S}+[\mu_{1}I+\mu_{2}I+\delta ](S^{1})+\mu_{1}SI\frac{E_{I}^{1}}{E_{I}}\\ & +[\nu_{1}+\delta ]\frac{{\left(E_{I}-E_{I}^{1}\right)}^{2}}{E_{I}}+[\nu_{1}+\delta ](E_{I}^{1})+\mu_{2}SI\frac{E_{U}^{1}}{E_{U}}+[\nu_{2}+\delta ]\frac{{\left(E_{U}-E_{U}^{1}\right)}^{2}}{E_{U}}\\ & +[\nu_{2}+\delta ](E_{U}^{1})+\nu_{1}E_{I}\frac{P^{1}}{P}+[\Pi_{1}+\delta ]\frac{{\left(P-P^{1}\right)}^{2}}{P}+[\Pi_{1}+\delta ](P^{1})+\nu_{2}E_{U}\frac{I^{1}}{I}\\ & +[\rho_{1}+\rho_{2}+\delta ]\frac{{\left(I-I^{1}\right)}^{2}}{I}+[\rho_{1}+\rho_{2}+\delta ](I^{1})+[\rho_{1}I+\tau_{1}T^{1}]\frac{A^{1}}{A}\\ & +[\omega_{1}+\delta ]\frac{{\left(A-A^{1}\right)}^{2}}{A}+[\omega_{1}+\delta ](A^{1})+\rho_{2}I^{1}\frac{T^{1}}{T}+[\tau_{1}+\delta ]\frac{{\left(T-T^{1}\right)}^{2}}{T}\\ & +[\tau_{1}+\delta ](T^{1})+\Pi_{1}P^{1}\frac{R^{1}}{R}+\delta \frac{R-R^{1}}{R}+\delta R^{1}. \end{array}$$

Since all the model parameters are positive, so CFDtαV(t)<0 when *q*_1_ < *q*_2_ and CFDtαV(t)=0 when *q*_1_ = *q*_2_. The case *q*_1_ = *q*_2_ implies that $S=S^{1},\;E_{I}=E_{I}^{1},\;E_{U}=E_{U}^{1},\;P=P^{1},\;I=I^{1},\;A=A^{1},\;T=T^{1},$ and *R* = *R*^1^. So, as per LaSalle’s invariant principle, the endemic equilibrium point $Q^{1}$ is GAS.□

## 5 Sensitivity analysis

Sensitivity analysis is used to identify the impact of the parameters on the dynamics of the disease. Numerous techniques for sensitivity analysis have been previously described and implemented for different epidemic models. In this manuscript, we used the elastic index, or normalized sensitivity index, as defined in [52] to compute the sensitivity index of a parameter *a*. The formula is given as follows:

$$\begin{array}{c} \begin{array}{cc} S_{a} & =\frac{a}{R_{0}}\frac{\partial R_{0}}{\partial a}. \end{array} \end{array}$$

**Fig 2:**
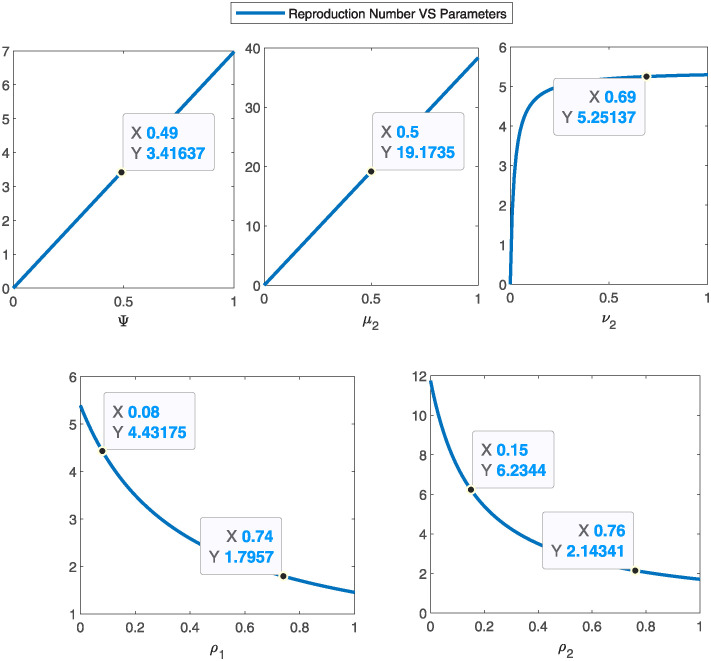
Sensitivity of the parameters against the selective model parameters. The increasing graphs show positive sensitivity index and the decreasing curves show the negative sensitivity index of the parameter to $R_{0}.$.

The sensitivity index of each parameter involved in reproduction number $R_{0}$ is given both analytically (see Table 1) and graphically (see Fig 2).

**Table 1:** Sensitivity index for $R_{0}:$ *μ*_2_ has a higher impact on the $R_{0}$. The *δ* and Ψ are the most sensitive parameters but both (birth and natural death rate) cannot be controlled. The force of interaction *μ*_2_ and treatment rate of the infectious *ρ*_2_ are the most positive sensitive parameters to $R_{0}$.

Parameters	Sensitivity Index	Parameters	Sensitivity Index
Ψ	1	*ρ*_1_	−0.2886836028
*μ*_2_	1	*ρ*_2_	−0.6735950732
*ν*_2_	0.2899408279	*δ*	−1.327662153

The parameter *μ*_2_ has a noteworthy impact on $R_{0}$, as indicated by the sensitivity index for $R_{0}$ (see Fig 2). Even though *δ* and Ψ show great sensitivity, they are inherently uncontrollable because they correspond to the birth and natural death rates, respectively. The rate at which the infection is transmitted, i.e., *ν*_2_, and the rate at which infectious individuals receive ART treatment, i.e., *ρ*_2_, also stand out as highly sensitive parameters, emphasizing their importance in controlling the basic reproduction number $R_{0}$. This analysis emphasizes the significance of concentrating on controllable parameters, such as interaction force and the disease transmission rate, directly or indirectly, to effectively manage and curtail the spread of infection.

## 6 Optimization design and analysis

In this section, we aim to formulate an optimal control problem to identify the most effective strategies for disease management. Initially, we update the disease model (7) by incorporating appropriate time-dependent controls. Subsequently, we define the cost functional to establish the optimization problem.

### 6.1 Updated model

**Fig 3:**
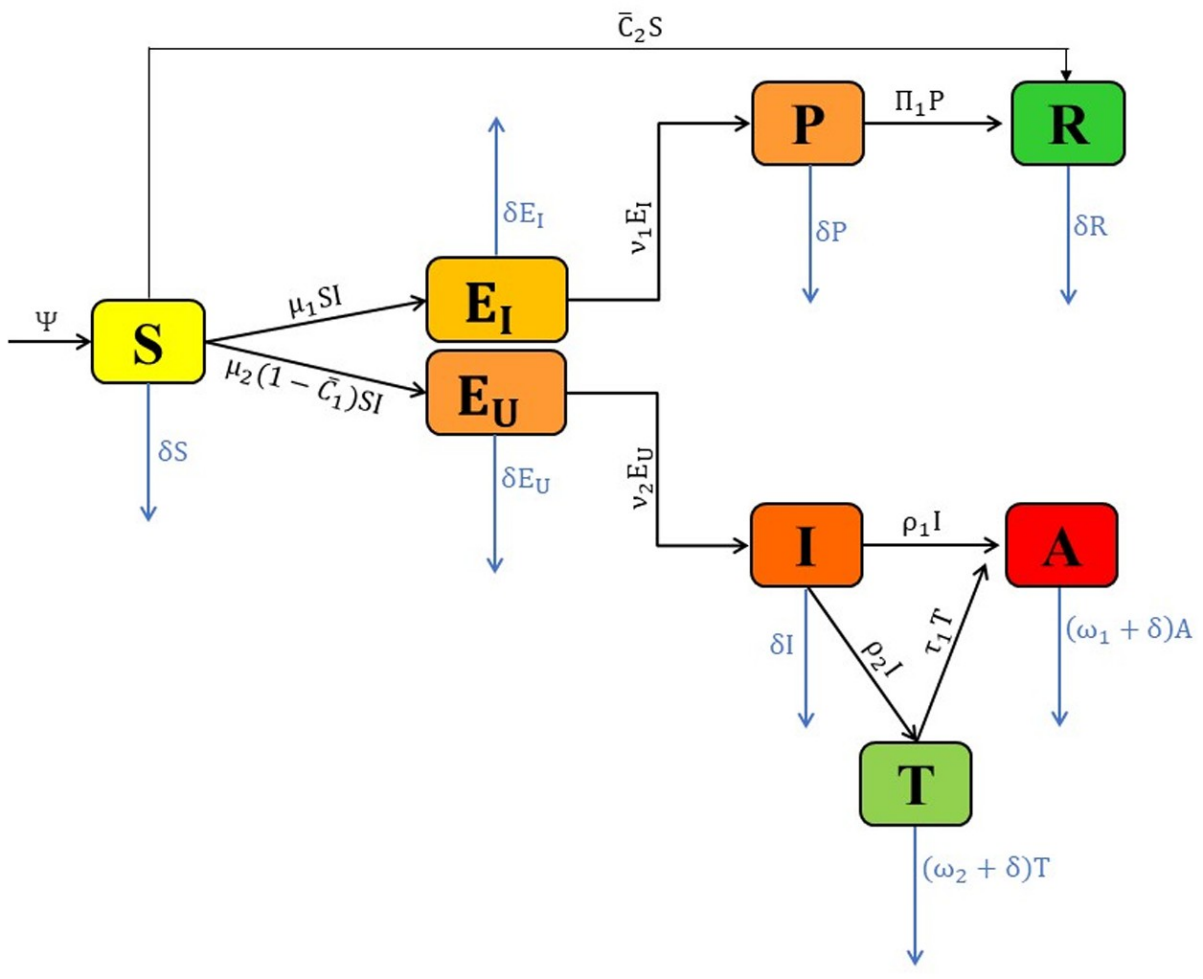
Flow diagram of the updated model: This figure is the updated form of the Fig 1 with controls $\bar{C_{1}}$ and $\bar{C_{2}}$ which represent the use of condoms and change in sexual habits, respectively.

HIV/AIDS can become a fatal disease if left untreated or if precautionary measures are neglected. We explored the crucial factors aiming to halt the spread of this disease. One of the primary methods is to implement safety precautions, such as the use of condoms and changes in sexual habits, to safeguard both the infectious individual and their partner against sexually transmitted diseases (STDs), i.e., HIV/AIDS. This constitutes a fundamental approach to preventing HIV/AIDS. Therefore, we update our proposed model with two additional time-dependent parameters: the use of condoms (${\bar{C}}_{1}\left(t\right)$) and the change in sexual habits (${\bar{C}}_{2}\left(t\right)$). With these considerations, the disease flow diagram is shown in Fig 3, and a mathematical representation of the updated flow diagram is given as follows:

CFDtαS(t)=Ψ-μ1S(t)I(t)-μ2(1-C¯1(t))S(t)I(t)-C¯2(t)S(t)-δS(t),CFDtαEI(t)=μ1S(t)I(t)-ν1EI(t)-δEI(t),CFDtαEU(t)=μ2(1-C¯1(t))S(t)I(t)-ν2EU(t)-δEU(t),CFDtαP(t)=ν1EI(t)-Π1P(t)-δP(t),CFDtαI(t)=ν2EU(t)-ρ1I(t)-ρ2I(t)-δI(t),CFDtαA(t)=ρ1I(t)+τ1T(t)-(ω1+δ)A(t),CFDtαT(t)=ρ2I(t)-τ1T(t)-(ω2+δ)T(t),CFDtαR(t)=C¯2(t)S(t)+Π1P(t)-δR(t), (26)

with ICs.

$$\begin{array}{c} S\left(0\right)>0,\;E_{I}\left(0\right)\geq 0,\;E_{U}\left(0\right)\geq 0,\;P_{0}\geq 0,\;I_{0}\geq 0,\;A_{0}\geq 0,\;T_{0}\geq 0,\;R_{0}\geq 0. \end{array}$$

The adjusted model (26) will serve as a constraint for the optimal control problem.

### 6.2 Optimal control problem

In this section, our objective is to mitigate the spread of the disease within a population. To achieve this goal, we aim to implement an optimal control strategy that effectively reduces the number of infectious individuals in the population at a minimal cost. To identify the appropriate optimal control for the given CF-fractional model (26), we employ Pontryagin’s Maximum Principle (PMP).

We consider the following cost functional to achieve the objectives of the study.

$$\begin{array}{c} W(E_{U},I,A,T,{\bar{C}}_{1},{\bar{C}}_{2})=\int_{0}^{T_{f}}[a_{1}E_{U}+a_{2}I+a_{3}A+a_{4}T+\frac{1}{2}w_{1}{\bar{C}}_{1}^{2}+\frac{1}{2}w_{2}{\bar{C}}_{2}^{2}]dt, \end{array}$$ (27)

where *E*_*U*_, *I*, *A*, and *T* are the state variables that represent the classes with infection; ${\bar{C}}_{1}$ and ${\bar{C}}_{2}$ are the time-dependent control variables; *T*_*f*_ is the final time; *a*_1_, *a*_2_, *a*_3_, and *a*_4_ are the weights of the state variables; and *w*_1_, *w*_2_ are the costs of controls.

To find the optimal strategies to control the disease at a minimum cost, the set of control variables is defined as:

$$\begin{array}{c} \bar{C}=\left\{\left({\bar{C}}_{1},{\bar{C}}_{2}\right)\;|\;{\bar{C}}_{i}\in \left[0,1\right],\;i=1,2\right\}. \end{array}$$

The objective is to find the controls ${\bar{C}}_{1}^{*},{\bar{C}}_{2}^{*}$ in the function space $\bar{C}$ such that the cost functional (27) is minimized, i.e.,

$$\begin{array}{c} J\left(E_{U},I,A,T,{\bar{C}}_{1}^{*},{\bar{C}}_{2}^{*}\right)=\min_{{\bar{C}}_{1},{\bar{C}}_{2}\in \bar{C}}J\left(E_{U},I,A,T,{\bar{C}}_{1},{\bar{C}}_{2}\right)\;\text{subject}\;\text{to}\;\text{the}\;\text{model}\;\text{(}26\text{)} \end{array}$$ (28)

We need to find the conditions that must be satisfied to find the optimal controllers for the fractional optimal control problem (28). For this, we define the Hamiltonian function H, given as follows:

$$\begin{array}{c} \begin{array}{cc} H(Z\left(t\right),\bar{C}\left(t\right),\eta \left(t\right))= & a_{1}E_{U}\left(t\right)+a_{2}I\left(t\right)+a_{3}A\left(t\right)+a_{4}T\left(t\right)+\frac{1}{2}w_{1}{\bar{C}}_{1}^{2}\left(t\right)+\frac{1}{2}w_{2}{\bar{C}}_{2}^{2}\left(t\right)\\ & +\eta_{1}(\Psi -\mu_{1}S\left(t\right)I\left(t\right)-\mu_{2}\left(1-{\bar{C}}_{1}\left(t\right)\right)S\left(t\right)I\left(t\right)-{\bar{C}}_{2}\left(t\right)S\left(t\right)-\delta S\left(t\right))\\ & +\eta_{2}(\mu_{1}S\left(t\right)I\left(t\right)-\nu_{1}E_{I}\left(t\right)-\delta E_{I}\left(t\right))\\ & +\eta_{3}(\mu_{2}\left(1-{\bar{C}}_{1}\left(t\right)\right)S\left(t\right)I\left(t\right)-\nu_{2}E_{U}\left(t\right)-\delta E_{U}\left(t\right))\\ & +\eta_{4}(\nu_{1}E_{I}\left(t\right)-\Pi_{1}P\left(t\right)-\delta P\left(t\right))\\ & +\eta_{5}(\nu_{2}E_{U}\left(t\right)-\rho_{1}I\left(t\right)-\rho_{2}I\left(t\right)-\delta I\left(t\right))\\ & +\eta_{6}(\rho_{1}I\left(t\right)+\tau_{1}T\left(t\right)-\left(\omega_{1}+\delta \right)A\left(t\right))\\ & +\eta_{7}(\rho_{2}I\left(t\right)-\tau_{1}T\left(t\right)-\left(\omega_{2}+\delta \right)T\left(t\right))\\ & +\eta_{8}({\bar{C}}_{2}\left(t\right)S\left(t\right)+\Pi_{1}P\left(t\right)-\delta R\left(t\right)), \end{array} \end{array}$$ (29)

where $Z\left(t\right),\;\bar{C}\left(t\right)$ and *η*(*t*) represent the state variables, control, and adjoint variables, respectively.

First optimal condition of PMP, i.e., $\frac{\partial H}{\partial \bar{C}}=0$ gives,

$$\begin{array}{c} \begin{array}{cc} \frac{\partial H}{\partial {\bar{C}}_{1}} & =0,\Rightarrow {\bar{C}}_{1}=\frac{\mu_{2}SI\left(\eta_{3}-\eta_{1}\right)}{w_{1}},\\ \frac{\partial H}{\partial {\bar{C}}_{2}} & =0,\Rightarrow {\bar{C}}_{2}=\frac{S(\eta_{1}-\eta_{8})}{w_{2}}. \end{array} \end{array}$$

Under max-min bounds, we have

$$\begin{array}{c} \begin{array}{cc} {\bar{C}}_{1}^{*} & =\min\{1,\text{max}\{0,\frac{\mu_{2}SI\left(\eta_{3}-\eta_{1}\right)}{w_{1}}\}\},\\ {\bar{C}}_{2}^{*} & =\min\{1,\text{max}\{0,\frac{S(\eta_{1}-\eta_{8})}{w_{2}}\}\}. \end{array} \end{array}$$ (30)

The second optimality condition of the PMP, -∂H∂Z=CFDtαηn(t), gives the following fractional order system of linear adjoint equations.

CFDtαη1(t)=-η1[μ1I+μ2I(1-C¯1)+C¯2+δ]+η2μ1I+η3μ2I(1-C¯1)+η8C¯2,CFDtαη2(t)=-η2(ν1+δ)+η4ν1,CFDtαη3(t)=-η3(ν2+δ)+η5ν2+a1,CFDtαη4(t)=-η4(Π1+δ)+η8Π1,CFDtαη5(t)=-η1[μ1S+μ2S(1-C¯1)]+η2μ1S+η3μ2S(1-C¯1)-η5(ρ1+ρ2+δ)+η6ρ1+η7ρ2+a2,CFDtαη6(t)=-η6(ω1+δ)+a3,CFDtαη7(t)=η6τ1-η7(τ1+ω2+δ)+a4,CFDtαη8(t)=-η8δ, (31)

with transversality conditions

$$\begin{array}{c} \eta_{i}\left(T_{f}\right)=0,\;i=1,\cdots ,8. \end{array}$$

Finally, the optimality condition CFDtαZi(t)=∂H∂ηi,i=1,…,8 gives us the state system (26).

To find the optimizer of the optimal control problem (28), we solve the optimality conditions (26), (30) and (31) by implementing steps of the following algorithm through MATLAB software.

**Algorithm 1**

*1. Set j* = 0 *and make a guess for control* ${\bar{C}}_{j}\in \bar{C}$.
*2. Solve the state system* (26) *and the corresponding adjoint system* (31) using ${\bar{C}}_{j}$.
*3. Calculate C*_*new*_ *by the process of categorization* (30).
*4. Revise control* ${\bar{C}}_{j}$ *by taking average of* ${\bar{C}}_{new}$ *and* ${\bar{C}}_{j}$ , *that is*, ${\bar{C}}_{j}=\frac{{\bar{C}}_{new}+{\bar{C}}_{j}}{2}$.
*5. **If*** ‖*θ*_*j*_ − *θ*_*j*−1_‖ < *tolerance*‖*θ_j_ for j* > 0 *STOP*
  **Otherwise** *j* → *j* + 1 and jump to step 2.

Here *θ* denotes each of the state variable $Z_{i}$, adjoint variable *η*_*i*_ and the control variable ${\bar{C}}_{j},\;j=1,2$. In step 5, tolerance is established for convergence of the solutions.

### 6.3 Solution approximating technique

To solve the CF fractional model (26), we adopt the fractional order Adams-Bashforth three-step technique [53]. The continuous time domain [0, *T*_*f*_] is transformed to *N* + 1 equal spaced discrete points, and the model is approximated at these points.

Application of the CF fractional integral to both sides of the compact model (26) yields the following integral solution:

$$\begin{array}{c} Z\left(t\right)=Z_{0}+\frac{2}{\left(2-\alpha )Ϝ(\alpha \right)}[\left(1-\alpha \right)G\left(Z\left(t\right)\right)+\alpha \int_{0}^{t}G\left(Z\left(s\right)\right)ds]. \end{array}$$

In discrete form:

$$\begin{array}{c} Z\left(t_{i}\right)=Z_{0}+\frac{2}{\left(2-\alpha )Ϝ(\alpha \right)}[\left(1-\alpha \right)G\left(Z\left(t_{i-1}\right)\right)+\alpha \int_{0}^{t_{i}}G\left(Z\left(s\right)\right)ds]. \end{array}$$ (32)

Similarly,

$$\begin{array}{c} Z\left(t_{i+1}\right)=Z_{0}+\frac{2}{\left(2-\alpha )Ϝ(\alpha \right)}[\left(1-\alpha \right)G\left(Z\left(t_{i}\right)\right)+\alpha \int_{0}^{t_{i+1}}G\left(Z\left(s\right)\right)ds]. \end{array}$$ (33)

Subtracting Eq (32) from (33), we get:

$$\begin{array}{cc} Z\left(t_{i+1}\right)-Z\left(t_{i}\right)= & \frac{2(1-\alpha )}{\left(2-\alpha )Ϝ(\alpha \right)}[G\left(Z\left(t_{i}\right)\right)-G\left(Z\left(t_{i-1}\right)\right)]+\frac{2\alpha }{\left(2-\alpha )Ϝ(\alpha \right)}[\int_{0}^{t_{i+1}}G\left(Z\left(s\right)\right)ds\\ & -\int_{0}^{t_{i}}G\left(Z\left(s\right)\right)ds]. \end{array}$$

Re-arranging the terms of the above equation, we reach at:

$$\begin{array}{c} Z\left(t_{i+1}\right)=Z\left(t_{i}\right)+\frac{2(1-\alpha )}{\left(2-\alpha )Ϝ(\alpha \right)}[G\left(Z\left(t_{i}\right)\right)-G\left(Z\left(t_{i-1}\right)\right)]+\frac{2\alpha }{\left(2-\alpha )Ϝ(\alpha \right)}[\int_{t_{i}}^{t_{i+1}}G\left(Z\left(s\right)\right)ds]. \end{array}$$ (34)

We approximate the integral of the Eq (34) by using the Lagrange interpolating polynomial of degree 2 and obtain the following finite difference approximation of the CF model (8).

$$\begin{array}{cc} Z(t_{i+1})= & Z\left(t_{i}\right)+\frac{2}{\left(2-\alpha )Ϝ(\alpha \right)}(1-\alpha +\frac{23}{12}h\alpha )G\left(Z_{i}\right)-\frac{2}{\left(2-\alpha )Ϝ(\alpha \right)}(1-\alpha +\frac{4}{3}h\alpha )G\left(Z_{i-1}\right)\\ & +\frac{10h\alpha }{12(2-\alpha )Ϝ(\alpha )}G\left(Z_{i-2}\right),\;i=2,\cdots ,n,\;\text{and}\;h=T_{f}/n. \end{array}$$ (35)

Note that if *α* = 1, the discrete Eq (35) becomes the trivial Adams-Bashforth three-step technique. For numerical simulations of disease model (7), we use values of parameters given in Table 2.

**Table 2:** Parametric values.

Parameters	Values	Reference	Parameters	Values	Reference
Ψ	0.55	[45]	*μ*_1_	0.1	Assumed
*μ*_2_	0.01/0.1	Assumed	*ν*_1_	0.05	Assumed
*ν*_2_	0.048	Assumed	*ρ*_1_	0.15	[46]
*ρ*_2_	0.35	[45]	Π_1_	0.04	Assumed
*τ*_1_	0.03	[46]	*ω*_1_	0.0909	[45]
*ω*_2_	0.0667	[46]	*δ*	0.0196	[45]

### 6.4 Analysis of optimal solutions

This section provides and discusses the optimal solution to the optimal control problem 28. The optimal solution is obtained by applying Algorithm 1 with the help of MATLAB code. The objective of the optimal control problem is to minimize the number of infected individuals and minimize the cost of control efforts. We implement the three-step Adams-Bashforth scheme, introduced by Owolabi and Atangana [54], to compute approximate solutions of CF fractional differential equations. The state and adjoint fractional equations are estimated using the Adams-Bashforth forward and backward schemes, respectively. The discretization of the scheme is explained in Section 6.3. It is initialized with an estimated value of the control effort during the stimulus time and then used to calculate the states and adjoints at each iteration using the Adams-Bashforth scheme (35). This process will continue until two sequential iterations of the state and adjoint become less than the predefined tolerance.

In the first case, we consider the use of condoms as a control parameter and analyze the effect of this control strategy on the dynamics of the HIV/AIDS disease, the objective function, and the cost of the control. As a second control strategy, we adopt change in sexual habits as a time-dependent control parameter and analyze the effect of the optimal control on the dynamics of the disease along with the objective functional. In the end, we use both optimal controls simultaneously and then analyze the impact of this control strategy. All control problems are computed with three different fraction orders, i.e., *α* = 0.6, 0.8, and 0.95. All the control strategies minimize the objective function and the spread of the disease. The possible outcome of each case is also discussed in the captions of each figure.

**Fig 4:**
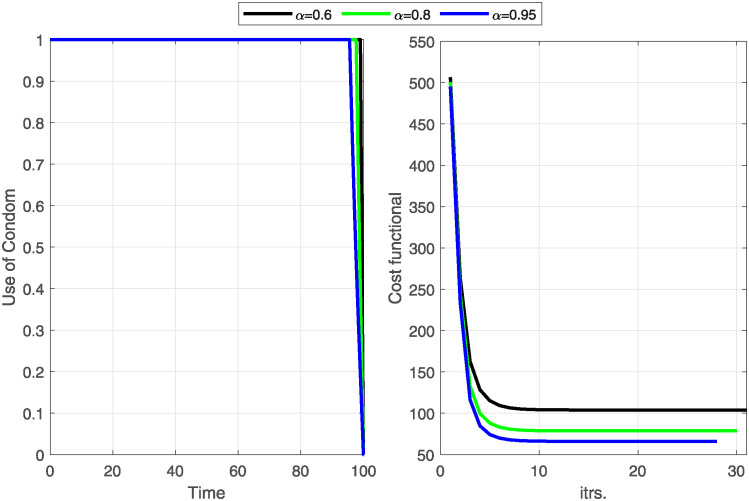
Cost functional and optimal control $\bar{C_{1}}$ (use of a condom) versus time. It is evident that the functional reaches its lowest under the optimal control in the 32^th^, 30^th^, and 28^th^ iterations for *α* = 0.6, *α* = 0.8, and *α* = 0.95, respectively. The optimal control rates are at maximum for the whole tenure and identify that to control the further spread of HIV/AIDS, the infectious population should always use condoms during sex.

**Fig 5:**
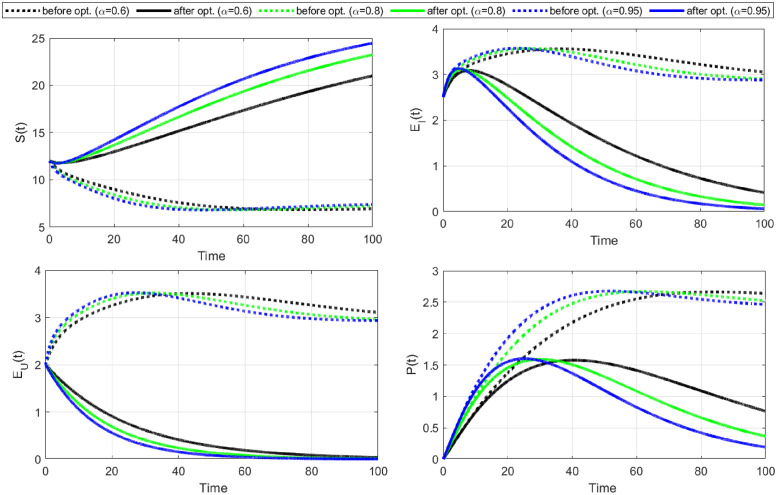
State variables versus time before (doted) and after (solid) optimization for $\bar{C_{1}}$. Susceptible individuals increase under the application of optimal control with each fractional-order derivative. A slight difference can be observed for different fractional orders. In addition, the number of both identified and unidentified exposed individuals decreased after optimal control.

**Fig 6:**
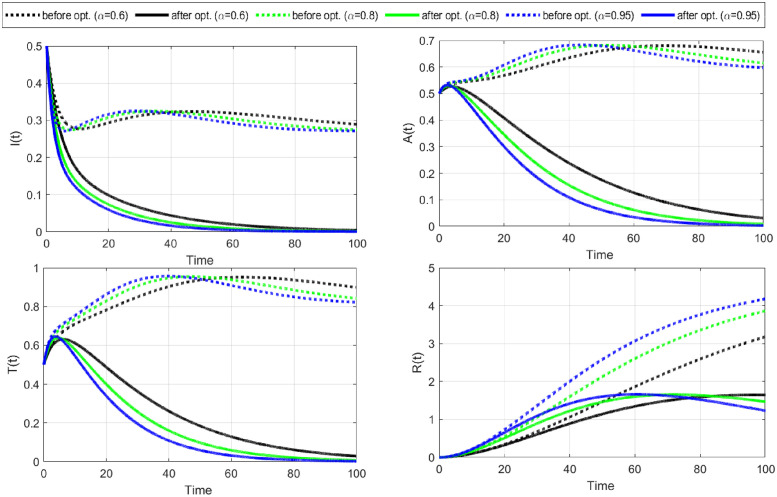
Continuations of Fig 5, showing that the number of infectious individuals in the population declines rapidly after optimal control $\bar{C_{1}}$. Furthermore, the number of people with full-blown AIDS goes to zero under the use of condoms as optimal control. Thus, a decrease in exposed and infectious population is the target of this optimal control strategy.

**case-1:** Firstly, we observe the application of condom strategy to HIV/AIDS disease control. We consider the control variable ${\bar{C}}_{1}$ as the only time-dependent control and take the cost of control *w*_2_ = 0. The numerical results for this strategy are shown in Figs 4–6.

Fig 4 illustrates the cost-functional and optimal control graph related to using condoms strategy. The data suggests that the functional reaches its minimum under optimal control at the 32^nd^, 30^th^, and 28^th^ iterations for *α* values of 0.6, 0.8, and 0.95, respectively. Notably, the objective functional achieves its lowest point in the fewest iterations when the fractional order is at its highest, which is *α* = 0.95. Throughout the entire period, the optimal control rates remain at their maximum, indicating that consistent condom use during sexual intercourse is imperative for effectively controlling the spread of HIV/AIDS. With no cure for HIV/AIDS currently available, maintaining safe sexual practices is crucial to prevent further transmission of the disease.

The optimal profiles of state variables before and after optimization $\bar{C_{1}}$ are shown in Figs 5 and 6. We notice that the number of susceptible individuals increases with the implementation of optimal control for each fractional-order derivative, with slight variations across different fractional orders. The number of identified and unidentified exposed individuals reduces substantially after applying optimal control. Additionally, the number of infectious individuals and those with full-blown AIDS also declines rapidly.

Our findings suggest that the policy ($\bar{C_{1}}$) effectively reduces the number of exposed and infectious individuals. However, it is crucial to acknowledge that the success of the policy depends on consistent and maximal condom use during sexual intercourse.

**Fig 7:**
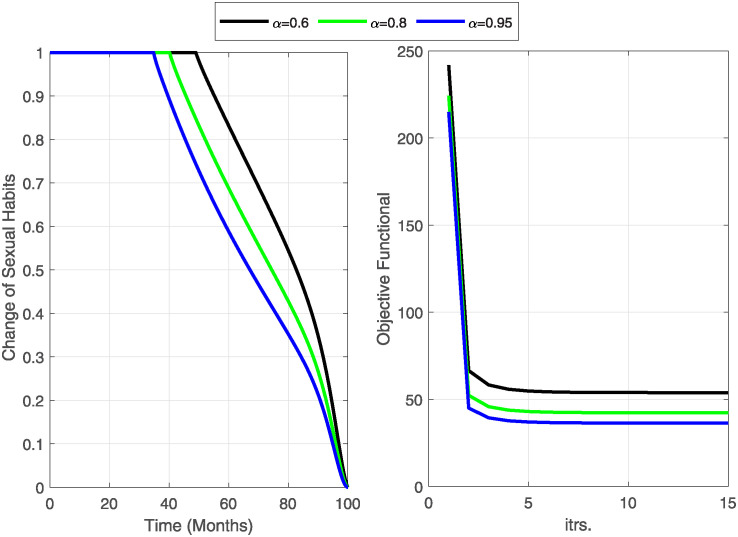
The objective functional and the behavior of the optimal control variable $\bar{C_{2}}$ (change in sexual habits) are given here. A change in sexual habits is also an effective strategy to control the spread of HIV/AIDS. It is also clear that a greater fractional order derivative is more suitable to minimize the objective functional and the cost of the control.

**Fig 8:**
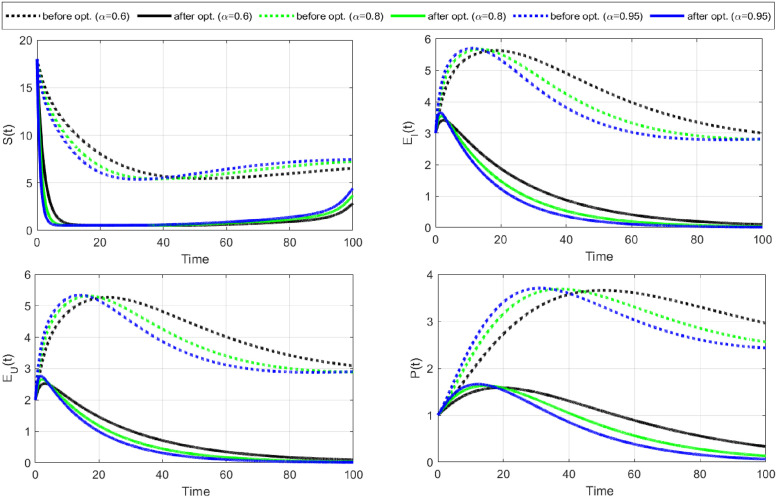
For Case-II, the system’s behavior is simulated with optimal control for $\bar{C_{2}}$, the rate at which susceptible individuals have changed and maintained their sexual habits and recovered. The number of susceptible people recovers without entering an infectious period more rapidly after optimization. Thus, the number of exposed individuals gradually decreases to zero.

**Case-II:** In this case, we study disease control under the application of changing sexual habits, such as avoiding injecting drugs, reducing the number of sexual partners, undergoing regular HIV testing, engaging in monogamous relationships, etc. To implement this strategy mathematically, we consider control variable ${\bar{C}}_{2}$ as the only time-dependent control and remove the cost of control $\bar{C_{1}}($ *w*_1_ = 0). The optimal solutions for this strategy are shown in Figs 7 and 8.

The objective functional and the behavior of the optimal control variable $\bar{C_{2}}$ (change in sexual habits) indicate that a higher fractional order derivative is more resourceful in minimizing the objective functional J and the relevant control cost (Fig 7). Notably, all fractional order derivatives achieve respective minima of cost functional within the same number of iterations, precisely fifteen. The strategy requires the support of individuals and healthcare providers to implement it a hundred percent for the first 50 days.

**Fig 9:**
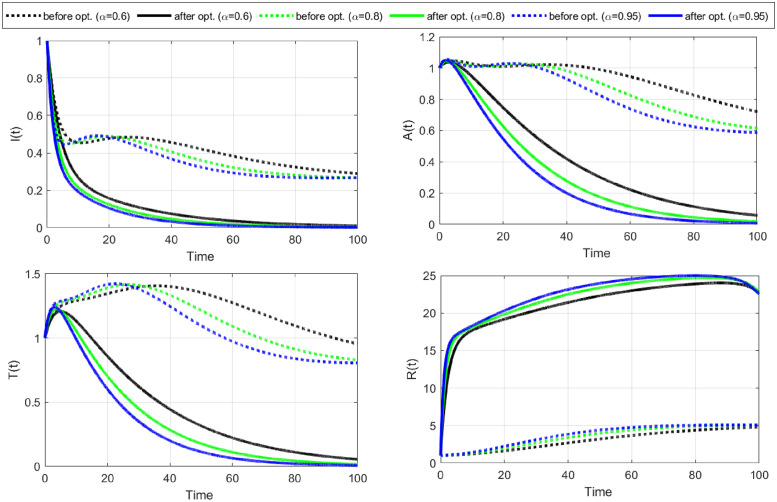
Continuous of Fig 8 Case-II. Infectious and later stages of the infection, i.e., fully developed AIDS (*A*(*t*)) and treatment (*T*(*t*)) are also decreased. However, the number of people who recovered grows gradually.

The profiles of state variables before and after optimization show that the number of susceptible individuals recovers without going to an infectious period after optimization (Fig 8). This is why we notice a great decline in susceptible individuals and a huge increase in recovered individuals after optimization. As a result, the number of exposed individuals gradually decreases to zero. Moreover, the number of people in infectious class *I*, fully developed AIDS class *A*, and treatment class *T* have also decreased to a minimum after optimization (Fig 9). These results illustrate that changing sexual habits is an effective approach to restricting the spread of HIV/AIDS and the above two approaches (Case-I& II) reveals that the strategy of changing sexual habits is more effective in restricting the spread of disease with a minimum cost of implementation.

**Fig 10:**
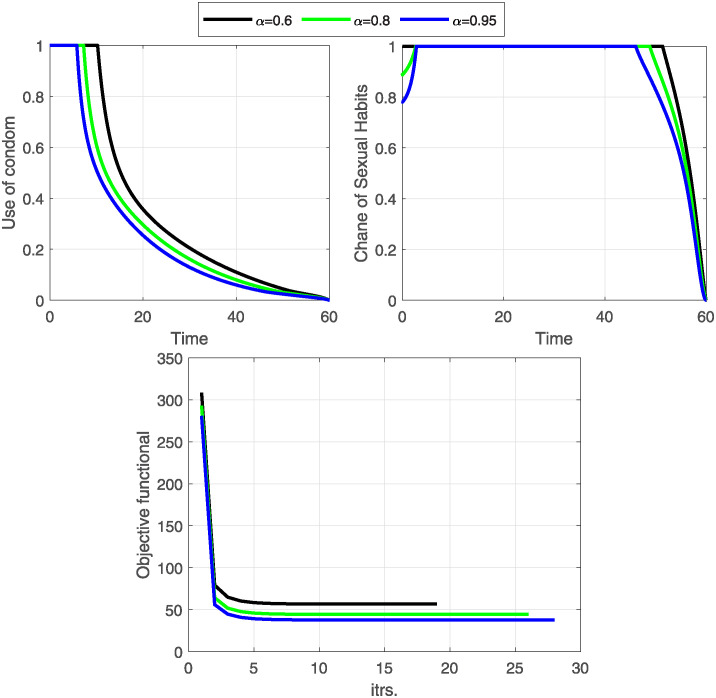
The objective functional is minimized under the influence of both optimal controls $\bar{C_{1}}\&\bar{C_{2}}$. The graph of the optimal controls shows that when a maximum number of susceptible individuals have changed their sexual habits, the burden of using condoms also decreases. Notably, an increase in fractional order increased the number of iterations but minimized the objective functional and the cost of the controls.

**Fig 11:**
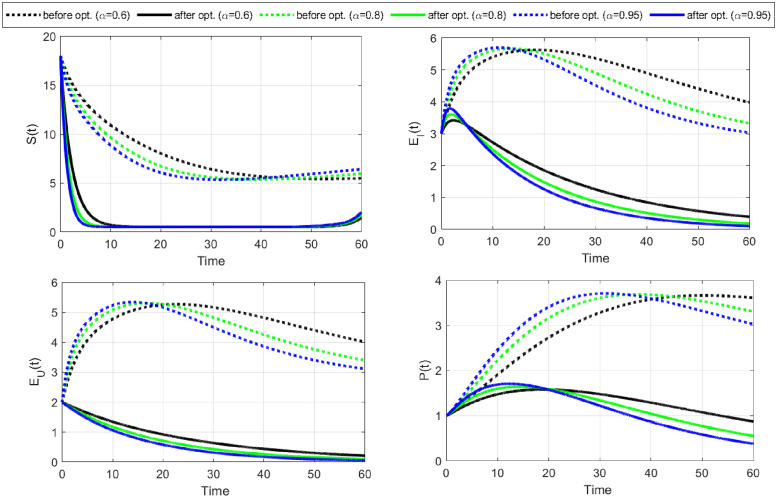
State variables before and after optimization of $\bar{C_{1}}\&\bar{C_{2}}$ (Case-III). The dynamics of the state variables decrease rapidly after optimization of change in sexual habits $\bar{C_{2}}$ and the use of condoms $\bar{C_{1}}$.

**Fig 12:**
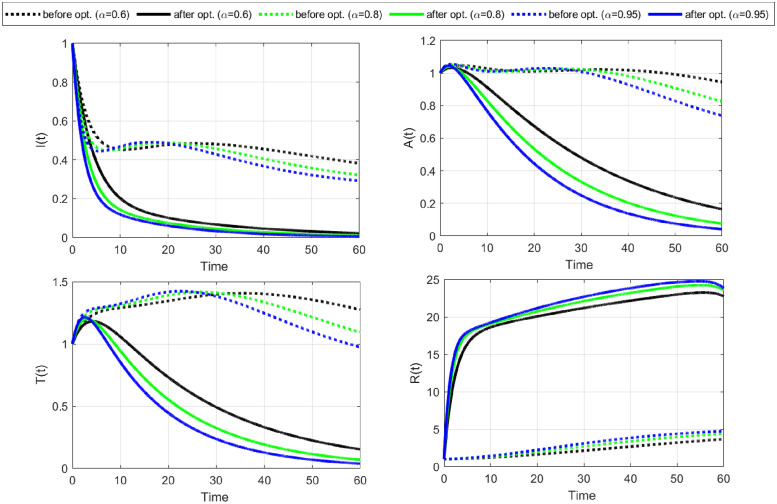
State variables before and after optimization for Case-III. The curves for state variables *I*, *A*, and *T* decrease gradually due to the change in sexual habits and the use of condoms. The increase in recovered individuals is largely due to the implementation of changing sexual habits.

**Case-III:** Lastly, we consider both controls ${\bar{C}}_{1},\;{\bar{C}}_{2}$ as time-dependent and comprehensively visualize their impact on disease dynamics and control costs. Numerical results of this strategy are shown in Figs 10–12.

The objective functional (Fig 10) has reached its minimum for each fractional order *α* under the influence of both optimal controls. The graphical representations of the optimal controls illustrate that as more susceptible individuals adopt safer sexual practices, the reliance on condom use decreases, leading to a reduction in associated burdens. It is important to note that while increasing the fractional order leads to more iterations, it effectively minimizes the objective functional and the overall control costs. This emphasizes the efficacy of employing higher fractional orders to achieve optimal outcomes in disease control.

The dynamics of state variables before and after optimization are shown in Figs 11 and 12. We again notice a significant decrease in identified and unidentified exposed individuals, infectious individuals, and those with fully developed AIDS. This decrease is largely due to the extensive adoption of changes in sexual habits. Additionally, there is a significant decrease in the number of susceptible individuals and a corresponding increase in the number of recovered ones. This shift highlights the effectiveness of behavioral interventions in controlling the spread of HIV/AIDS and facilitating recovery.

The implementation of all three strategies effectively limits the spread of HIV/AIDS within the population. A comparative analysis of the three cases shows that practicing safe sexual habits (changing sexual habits) is the most cost-effective strategy for controlling the disease. This approach not only reduces the transmission of HIV/AIDS but also minimizes the associated control costs, demonstrating its superiority over other methods.

## 7 Conclusion

In this study, we developed a novel CF-fractional model to enhance the understanding of HIV/AIDS dynamics and propose effective disease-control strategies. We analyzed the proposed model for the existence of a unique, positive, and bounded solution. Moreover, we determined the reproduction number $R_{0}$ and explored the model for stability analysis. We demonstrated the local and global stability of the model at disease-free and endemic equilibrium points. Establishing and comprehending these fundamental stability concepts is pivotal for accurately forecasting the progression of an epidemic and formulating effective control strategies.

Through sensitivity analysis, we identified the transmission rate *μ*_2_ as a parameter with a significant impact on $R_{0}$, emphasizing its effectiveness in disease control. Leveraging these findings, we updated our proposed model and demonstrated its efficacy in reducing HIV/AIDS transmission by controlling sexual contact rates and modifying sexual habits among susceptible individuals. We introduced a cost-functional and defined an optimal control problem to minimize the number of exposed, unidentified, infected, fully blown AIDS and treated individuals while reducing costs. We used Pontryagin’s maximum principle to derive optimality conditions and solved them numerically by implementing steps of the solution algorithm.

Our study investigated three distinct optimal control approaches: solely using condoms, solely changing sexual habits, and a combined approach involving both condom use and changing sexual habits. The graphical results demonstrated the efficacy of each approach in reducing HIV infections and minimizing implementation costs. Our findings highlight the simplicity and effectiveness of strategies that target interactions between susceptible and infectious individuals and promote safer sexual habits among susceptible individuals to prevent and mitigate the disease. After comparing the three strategies, it appears that promoting safe sexual practices is the most effective approach for disease control. This approach not only reduces HIV/AIDS transmission but also minimizes associated control costs, establishing its superiority over the other two considered methods.

The findings of this research have substantial implications for public health policy. The study emphasizes the necessity of implementing early intervention strategies to slow the progression of the disease. Additionally, the study highlights the effectiveness of encouraging safe sexual practices and behavioral changes, which are both cost-effective and adaptable to various cultural contexts for the control of HIV/AIDS. Policymakers can utilize these insights to formulate educational campaigns, subsidize the provision of condoms, and support community-based initiatives aimed at vulnerable populations.

This study establishes a basis for future research in infectious disease modeling by integrating fractional-order dynamics with optimal control strategies. It includes cost-functional analysis to consider economic factors in disease management. Researchers can further refine the model for multi-strain diseases and include other components like pre-exposure prophylaxis (PrEP) for a deeper understanding of disease dynamics.
